# Publishing data to support the fight against human vector-borne diseases

**DOI:** 10.1093/gigascience/giac114

**Published:** 2022-11-03

**Authors:** Scott C Edmunds, Florence Fouque, Kyle A Copas, Tim Hirsch, Paloma Helena Fernandes Shimabukuro, José Dilermando Andrade-filho, Catalina Marceló, Carlos Andrés Morales, María Camila Lesmes, Patricia Fuya, Sergio Méndez, Horacio Cadena, Álvaro Ávila-Díaz, Erika Santamaría, Živko Južnič-Zonta, Roger Eritja, John R B Palmer, Frederic Bartumeus, Maurício dos Santos-Conceição, Samira Chahad-Ehlers, Cássio Lázaro Silva-Inácio, Ana Leuch Lozovei, Andrey José de Andrade, Sara Paull, Miguel Ángel Miranda, Carlos Barceló, Francis Schaffner, Alessandra Della-Torre, Dimitri Brosens, Wouter Dekoninck, Guy Hendrickx, Wim Van Bortel, Isra Deblauwe, Nathalie Smitz, Veerle Versteirt, Rodrigo Espindola Godoy, Andreia Fernandes Brilhante, Soledad Ceccarelli, Agustín Balsalobre, María Eugenia Vicente, Rachel Curtis-Robles, Sarah A Hamer, José Manuel Ayala Landa, Jorge E Rabinovich, Gerardo A Marti, Dmitry Schigel

**Affiliations:** GigaScience Press, BGI Hong Kong Tech Co Ltd., RmA 26F Kings Wing Plaza 2, 1 On Kwan Street, Shek Mun, Sha Tin, NT, Hong Kong SAR; Special Programme for Research & Training in Tropical Diseases (TDR), World Health Organization, Avenu Appia 20, 1211 Geneva 27, Switzerland; GBIF Secretariat Universitetsparken 15. DK-2100 Copenhagen Ø, Denmark; GBIF Secretariat Universitetsparken 15. DK-2100 Copenhagen Ø, Denmark; Coleção de Flebotomíneos (FIOCRUZ/COLFLEB), Instituto René Rachou, Fiocruz Minas Avenida Augusto de Lima, 1715 - Barro Preto, 30190009, Belo Horizonte, Brazil; Grupo de Estudos em Leishmanioses, Instituto René Rachou, Fiocruz Minas Avenida Augusto de Lima, 1715 - Barro Preto, 30190009, Belo Horizonte, Brazil; Coleção de Flebotomíneos (FIOCRUZ/COLFLEB), Instituto René Rachou, Fiocruz Minas Avenida Augusto de Lima, 1715 - Barro Preto, 30190009, Belo Horizonte, Brazil; Grupo de Estudos em Leishmanioses, Instituto René Rachou, Fiocruz Minas Avenida Augusto de Lima, 1715 - Barro Preto, 30190009, Belo Horizonte, Brazil; Grupo de Entomología, Instituto Nacional de Salud, 111321, Bogotá, Colombia; Secretaría de Salud Departamental del Cauca, 190003, Popayán, Colombia; Grupo de Entomología, Instituto Nacional de Salud, 111321, Bogotá, Colombia; Universidad de Ciencias Aplicadas y Ambientales, 111166, Bogotá, Colombia; Grupo de Entomología, Instituto Nacional de Salud, 111321, Bogotá, Colombia; Grupo de Entomología, Instituto Nacional de Salud, 111321, Bogotá, Colombia; Programa de Estudio y Control de Enfermedades Tropicales PECET, 050010, Medellín, Colombia; Universidad de Ciencias Aplicadas y Ambientales, 111166, Bogotá, Colombia; Grupo de Entomología, Instituto Nacional de Salud, 111321, Bogotá, Colombia; Centre d'Estudis Avançats de Blanes (CEAB-CSIC), C/d'accés a la Cala St. Francesc 14, 17300 Blanes, Girona, SpainCentre d'Estudis Avançats de Blanes (CEAB-CSIC), C/d'accés a la Cala St. Francesc 14, 17300 Blanes, Girona, Spain; Centre de Recerca Ecològica i Aplicacions Forestals (CREAF), Edifici C Campus de, 08193 Bellaterra, Barcelona, Spain; Departament de Ciències Polítiques i Socials, Universitat Pompeu Fabra, Plaça de la Mercè, 10-12, 08002 Barcelona, Spain; Centre d'Estudis Avançats de Blanes (CEAB-CSIC), C/d'accés a la Cala St. Francesc 14, 17300 Blanes, Girona, SpainCentre d'Estudis Avançats de Blanes (CEAB-CSIC), C/d'accés a la Cala St. Francesc 14, 17300 Blanes, Girona, Spain; Centre de Recerca Ecològica i Aplicacions Forestals (CREAF), Edifici C Campus de, 08193 Bellaterra, Barcelona, Spain; Institució Catalana de Recerca i Estudis Avançats (ICREA), 23 Passeig de Lluís Companys, 08010 Barcelona, Spain; Basic Pathology Department, Federal University of Paraná, Av. Cel. Francisco H. dos Santos, 100 - Jardim das Américas, Curitiba, PR 81531-980, Brazil; Genetics and Evolution Department, Federal University of São Carlos, Rodovia Washington Luís, km 235 SP-310, São Carlos, SP 13565-905, Brazil; Microbiology and Parasitology Department, Federal University of Rio Grande do Norte, Av. Senador Salgado Filho, 3000, Natal, RN 59078-970, Brazil; Basic Pathology Department, Federal University of Paraná, Av. Cel. Francisco H. dos Santos, 100 - Jardim das Américas, Curitiba, PR 81531-980, Brazil; Post-graduate Programme in Entomology, Zoology Department, Federal University of Paraná, Av. Cel. Francisco H. dos Santos, 100 - Jardim das Américas, Curitiba, PR 81531-980, Brazil; National Ecological Observatory Network, Battelle, 1685 38^th^ St, Boulder, CO 80301, USA; Applied Zoology and Animal Conservation group, University of the Balearic Islands (UIB), Ctra Valldemossa km 7.5, 07122 Palma, Spain; Applied Zoology and Animal Conservation group, University of the Balearic Islands (UIB), Ctra Valldemossa km 7.5, 07122 Palma, Spain; Francis Schaffner Consultancy, Lörracherstrasse 50, 4125 Riehen, Switzerland; Dep. Public Health and Infectious diseases, University Sapienza, Piazzale Aldo Moro 5, 00185 Roma, Italy; Research Institute for Nature and Forest (INBO), Havenlaan 88 b73, 1000, Brussels, Belgium; Royal Belgian Institute for Natural Sciences (RBINS - BopCo & Scientific Heritage Service), Vautierstraat 29, 1000, Brussels, Belgium; Avia-GIS NV, Risschotlei 33, 2980, Zoersel, Belgium; Unit Entomology, Dept. of Biomedical Sciences, Institute of Tropical Medicine (ITG), Nationalestraat, 155, 2000, Antwerpen, Belgium; Unit Entomology, Dept. of Biomedical Sciences, Institute of Tropical Medicine (ITG), Nationalestraat, 155, 2000, Antwerpen, Belgium; Royal Museum for Central Africa (RMCA - BopCo), Leuvensesteenweg 17, 3080 Tervuren, Belgium; Agency for Nature and Forests, (ANB), Havenlaan 88 b75, 1000, Brussels, Belgium; Independent Researcher, Brazil; Universidade Federal do Acre, Departamento de Ciências da Saúde e Educação Física. Universidade Federal do Acre, Distrito Industrial, Rio Branco, 69920900, Er, Brasil; Centro de Estudios Parasitológicos y de Vectores (CEPAVE-CCT-La Plata-CONICET-UNLP), La Plata, Buenos Aires 1900, Argentina; Consejo Nacional de Investigaciones Científicas y Técnicas (CONICET), Buenos Aires, 1002, Argentina; Centro de Estudios Parasitológicos y de Vectores (CEPAVE-CCT-La Plata-CONICET-UNLP), La Plata, Buenos Aires 1900, Argentina; Consejo Nacional de Investigaciones Científicas y Técnicas (CONICET), Buenos Aires, 1002, Argentina; Centro de Estudios Parasitológicos y de Vectores (CEPAVE-CCT-La Plata-CONICET-UNLP), La Plata, Buenos Aires 1900, Argentina; College of Veterinary Medicine and Biomedical Sciences, Texas A&M University, College Station, Texas, 77845, USA; College of Veterinary Medicine and Biomedical Sciences, Texas A&M University, College Station, Texas, 77845, USA; Facultad de Agronomia, UCV, Apdo. 4579, Museo del Instituto de Zoología Agrícola (MIZA), 2101A, Maracay, Venezuela; Centro de Estudios Parasitológicos y de Vectores (CEPAVE-CCT-La Plata-CONICET-UNLP), La Plata, Buenos Aires 1900, Argentina; Consejo Nacional de Investigaciones Científicas y Técnicas (CONICET), Buenos Aires, 1002, Argentina; Centro de Estudios Parasitológicos y de Vectores (CEPAVE-CCT-La Plata-CONICET-UNLP), La Plata, Buenos Aires 1900, Argentina; Consejo Nacional de Investigaciones Científicas y Técnicas (CONICET), Buenos Aires, 1002, Argentina; GBIF Secretariat Universitetsparken 15. DK-2100 Copenhagen Ø, Denmark

## Abstract

Vector-borne diseases are responsible for more than 17% of human cases of infectious diseases. In most situations, effective control of debilitating and deadly vector-bone diseases (VBDs), such as malaria, dengue, chikungunya, yellow fever, Zika and Chagas requires up-to-date, robust and comprehensive information on the presence, diversity, ecology, bionomics and geographic spread of the organisms that carry and transmit the infectious agents. Huge gaps exist in the information related to these vectors, creating an essential need for campaigns to mobilise and share data. The publication of data papers is an effective tool for overcoming this challenge. These peer-reviewed articles provide scholarly credit for researchers whose vital work of assembling and publishing well-described, properly-formatted datasets often fails to receive appropriate recognition. To address this, *GigaScience*’s sister journal *GigaByte* partnered with the Global Biodiversity Information Facility (GBIF) to publish a series of data papers, with support from the Special Programme for Research and Training in Tropical Diseases (TDR), hosted by the World Health Organisation (WHO). Here we outline the initial results of this targeted approach to sharing data and describe its importance for controlling VBDs and improving public health.

## Body Text

Free and open access to biodiversity data enables research and analysis needed to confront the threats and growing burden that vector-borne diseases and their control place on human health.

The World Health Organization (WHO)’s Global Vector Control Response (GVCR) 2017–2030 calls for additional efforts on data sharing on disease vectors, and Pillar 3 of the GVCR emphasizes vector surveillance and monitoring of interventions. Universal, free and open access to data on vectors will help countries to strengthen their response against vector-borne diseases and to improve health and well-being. The achievement of this goal requires development and management of databases within accessible platforms where all stakeholders, from researchers to those implementing vector control, can find supplementary information and experience. TDR, the special program for research and training on tropical diseases, hosted by WHO, is committed to helping researchers, especially from low and lower-middle income countries (LMICs), to have access to data-sharing platforms and to enhance their capacity to publish their data and thus make them accessible to the wider community. Data papers are a cost-efficient and effective tool to increase digital availability of relevant biodiversity data, and to mainstream data openness across research communities. Support of data papers on disease vectors through sponsorship of special issues is thus fully aligned with TDR's objectives.

The Global Biodiversity Information Facility (GBIF) has in recent years identified a number of priority research and policy areas where increased availability of biodiversity data would provide a richer evidence base. These include data-intensive, cross-disciplinary research into vectors and reservoirs of human diseases, for which critical taxonomic and geographic data gaps on the occurrence of species represent a significant obstacle. In 2020, GBIF formed a task group on mobilization and use of biodiversity data for research and policy on human diseases, with a mandate to provide advice, priority directions and expert opinions. Among the measures supported by this task group is promotion of and support for the publication of data papers, as a means of encouraging data sharing by a community of data holders largely unfamiliar with the process of publishing biodiversity datasets through GBIF. As data papers ideally describe well-prepared datasets already available in a platform such as GBIF, sponsored calls for submissions with article processing charges (APCs) waived for authors, provide a direct incentive and support for publishing datasets that are correctly formatted and of high quality. With support from TDR a first sponsored call for human disease vector data papers in *GigaByte* journal was announced in November 2021, and after going through peer review the resulting first phase of papers were all published by the end of May 2022 [1]. Many biodiversity data experts within the GBIF collaborative network, as well as task group members, provided direct support for preparation of the data papers submitted to *GigaByte*, helping to bridge the domains of biodiversity and biomedical research. Following positive reaction to these activities within the GBIF network and from TDR, plans are under development to scale up this initial experiment across taxa, diseases, regions and complexity of biological systems. And a second call for papers for the *GigaByte* series has also just been reopened.

As well as being on hand for any papers that required curation and hosting of large supplemental files, the GigaScience Press GigaDB team were also on hand to assist the data peer review process. This involved data auditing and providing a data review for each submission, ensuring the data was open and FAIR (findable, accessible, interoperable and reusable). The review included selecting a number of data points to be carefully inspected, and verifying that the total number of occurrences and the geographic range were consistent with the details in the paper. To comply with *GigaByte*’s stringent open data policies, the journal insisted upon use of CC0 public domain waivers for the datasets described in the data papers. In some of the submissions, the review also picked up inconsistencies in the metadata caused by conversion problems and non-ASCI characters. In line with Open Peer Review, the peer reviews and data review templates are available for scrutiny via the Article Review History tabs on all of the papers.

**Figure 1:**
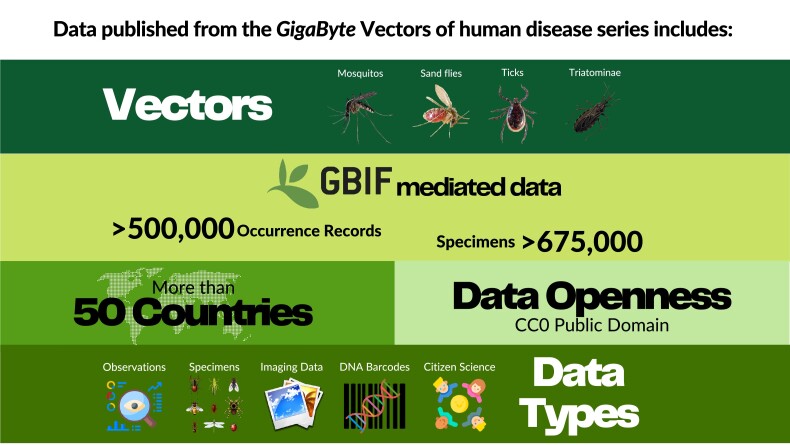
A summary of the disease vector data shared through the first phase of the sponsored call for data papers.

The results of this first call for data papers have been promising and the results are highlighted here. The first 11 publications present more than 500 000 occurrence records linked to 675 000 specimens across over 50 countries (Fig. 1) [1]. The data described in the papers includes occurrence records (including specimen collections) published through GBIF.org, imaging data in the EBI Bioimaging repository, DNA barcodes in NCBI, and other miscellaneous data hosted in GigaDB and Dryad repositories. Publication in *GigaScience*’s sister journal *GigaByte* has enabled use of a new state-of-the-art XML-first publishing platform, and the papers include embedded dynamics, such as interactive maps and embedded protocols. Additionally, papers are linked to preprints and there are multilingual options for many papers that allow Portuguese and Spanish speakers to better understand the implications of important work relating to the public health of their communities. In the next section we outline this first phase of submissions, what they presented, and what has been learned from the process of publishing them.

## Important stories that can be told through open data

As well as mobilizing a significant volume of data, the submissions represented diverse vectors, locations and forms. And while the datasets are presented primarily for re-use by others, an analysis reveals several interesting insights from the data itself.

The online catalogue of the Coleção de Flebotomíneos (FIOCRUZ/COLFLEB) has been derived from approximately 72 000 individual specimens and 370 species deposited at the René Rachou/Fiocruz Institute in Belo Horizonte, Brazil [2]. This dataset covers over 80 years of sandfly research in Brazil and 20 countries in the Americas, making it the largest and most comprehensive collection of these insects. Approaches to capturing this data has varied, with another paper also digitising observations of all published scientific studies on sandflies in the Brazilian state of Acre [3].

As well as national and international scale collections, papers also presented work collected in the lands of indigenous peoples in the Brazilian Amazon [4]. These sandfly vector records were obtained from areas of disease transmission where cutaneous leishmaniasis is endemic, and has grown with changes in the environment and hunting practices. The authors hope that these records will contribute to a better understanding of leishmaniasis transmission dynamics among these communities, as well as to increase data on the distribution of these insect vectors in locations that are remote and difficult to access, and that therefore are surveyed by public health systems.

The “Ana Leuch Lozovei” collection presents an incredible diversity of 100 species of Culicidae in 18 municipalities in Paraná state, Southern Brazil, collected between 1967 and 1999 [5]. It records three species for the first time in Brazil, signifying the expansion of geographical distribution of the species previously restricted to certain locations or countries.

The public health importance of this data has been very clear, such as a collection of data coming from the screening of urban households in three municipalities with a high incidence of dengue in Southwestern Colombia [6]. It presents novel data for the geographical distribution of 2383 specimens belonging to the Culicidae family, alongside the house infestation percentage per municipality and additional descriptive measures of the sampled mosquitos at each location. This type of data is not often reported due to the sampling effort involved in entomological sampling.

The MODRISK [7] and MEMO [8] projects presented the outputs of state-of-of-the-art monitoring of the exotic *Aedes* genus in Belgium. MODRISK uses a novel randomised approach to model mosquito biodiversity distribution at a 1-km resolution, based on longitudinal data, systematic screens and historical collections going back to 1878. MEMO (Monitoring of Exotic MOsquitoes in Belgium) looks at early detection of exotic mosquito species along high-risk introduction routes in Belgium, where data is collected at defined points of entry. It also includes genetic sequencing data, as DNA-barcoding was used as a quality control step to validate 5% of the morphological identifications. This work showed that new exotic species could even be detected in temperate Belgium, such as *Ochlerotatus/Aedes koreicus*, which had not been reported in Europe until then.

In addition to national surveillance schemes, collaborative supranational projects are also represented in the series, with AIMSurv presenting data from the first pan-European harmonized surveillance of *Aedes* invasive mosquito species organized under the framework of the AIMCOST Action [9]. In 2020, AIMSurv was implemented by 42 teams from 24 countries. Data comprised a core file with 19 130 samples that improve knowledge of the European seasonal pattern of the Aedes invasive mosquito species *Ae. albopictus, Ae. japonicus* and *Ae. koreicus*.

Citizen scientists are accounted for, with the Mosquito Alert dataset including occurrence records of adult mosquitoes collected by citizens through the Mosquito Alert smartphone app [10]. Each record is linked to a photograph which is validated by entomological experts to assess the species. The paper shows that citizens can be part of a mature near-real-time surveillance system of targeted disease-vector mosquito species of concern in the EU. From a surveillance perspective, the system has been able to detect many appearances of *Aedes albopictus* well beyond its immediate expansion front. Another major highlight is the first detection in 2018 of *Aedes japonicus* in Spain, an isolated population located 1300 km away from its previously nearest known location in Europe.

Another project that includes citizen-collected data forms a sub-dataset of American triatomine, insect vectors involved in Chagas disease that are also known as “kissing bugs” [11]. With 90% of the US collected data obtained from the Kissing bugs and Chagas Disease in the United States community science program. The work is the result of an exhaustive review of public information combined with substantial inter-institutional collaboration, which integrated information spanning 24 countries in the Americas.

Other vectors include work presenting abundance, diversity and pathogen data on ticks, collected by the United States National Ecological Observatory Network (NEON) [12]. The dataset is unique because of the availability of detailed surveillance data alongside tick pathogen infection data across a large geographic area (428 960 occurrences across the whole United States including Alaska and Puerto Rico). An unprecedented level of associated environmental data collected at the same sites is also available from the NEON data portal, including: remote sensing data, data on abiotic variables, and observational data on nearby flora and fauna. This makes it optimal for complex and multi-scale analyses of changing tick distributions, tick invasions and the patterns and processes underlying tick vector, host and pathogen dynamics. Archived samples of ticks and genomic extracts included in the dataset add to its value and re-use potential.

## Lessons learned through data publication

Polling the many authors of these papers for what they have learned from producing and publishing these data papers, the most common responses were how to prepare a data paper understanding the GBIF publication process and maximising their data collection´s availability to the wider public. Authors highlighted the high level of collaboration among all participants, since the activity was based on free collaboration and data sharing. Data papers can also provide proper recognition of large numbers of participants that can contribute to data collection in the form of author and/or consortium collaborators (e.g., AIMSurv, with 78 authors and 92 collaborators). Collectively, free and open access to this data on biodiversity enables its sharing and integration with other vector surveillance initiatives around the world. As well as their own learning, the authors gained experience that also enabled them to “teach/advocate” to their co-authors and collaborators about open data, open science, data management, standards and the data publication process. Mapping datasets to Darwin Core terms provided new insight into the contents of these datasets and linkages between vectors and pathogens. Providing a succinct overview of the utility of the dataset and the data collection and quality control methods helps data producers to better communicate the strength of the data and potential questions for analysis.

As much of the work was based on historical collections and reviews of literature, the authors found the process of putting these papers together enlightening to understand the chronology and evolution in the knowledge of the diversity of these vectors in the regions studied. They also highlighted the geographical and temporal data gaps still remaining.

Some of the authors feel the data papers may turn out to become even more important than analysis papers. Since data is the driving force of science and should always be made freely available for any party to work with, writing and openly sharing these data papers contributes to that essential idea.

The process of writing these papers also provoked very important questions regarding the nature of these types of collaborations, bringing together entomologists, public health researchers, data experts, and even citizens and independent researchers not usually credited for their contributions to scientific publications.

The Mosquito Alert team had to make many decisions on how to put together an organic, ever growing and mutating data collection system with many actors involved, and highly heterogeneous contributions to the dataset by experts. They worked to come up with the right format for author citation, and to set up a credit system to evaluate contributions from multiple and diverse collaborators. This exercise triggered many debates within the Mosquito Alert team, and between the Mosquito Alert team and the expert validation community, on issues going much beyond the dataset itself. The authors these efforts represent a big step towards the development of a system to give credit to the worldwide community of digital entomologists contributing to the Mosquito Alert dataset, and to better application of the FAIR principles which they aim to follow in the future. This has helped the Mosquito Alert team with their ongoing efforts, and also provides a model for other projects involving diverse collaborations and citizen science to share their datasets.

## Conclusions

Free and open access to easily discoverable data are key to making rapid advances in all areas of research. Opening this call has led directly to researchers and institutions sharing a wealth of data that is of extremely important public health interest. These collections represent potential reference for possible changes in the prevalence and geographic distribution of vector species for future entomological surveys. With the series now open for a second phase of submissions, this approach will continue to fill not just the remaining taxonomic and geographic gaps, but as an ongoing project hopefully also temporal ones as well.

## Additional Files

**Supplementary File 1**. Interview questions with the vectors of human disease series authors.

giac114_Supplemental_File

## Data Availability

The associated papers and datasets linked to them are all listed in the supplemental file (Supplementary 1).

## Editor's Note

In part due to the novel publishing process, features, and outputs from this series, *GigaByte* was the winner of the 2022 Association of Learned and Professional Society Publishers (ALPSP) Innovation in Publishing Award. *GigaByte* is now taking submissions for the second round for this series with sponsored APCs. Please contact editorial@gigabytejournal.com if you have any potential submissions or questions.

## Abbreviations

APCs: article processing charges; GBIF: Global Biodiversity Information Facility; GVCR: Global Vector Control Response; MEMO: Monitoring of Exotic MOsquitoes in Belgium; NEON: National Ecological Observatory Network; TDR: Special Programme for Research and Training in Tropical Diseases, VBD: vector-bone diseases; WHO: World Health Organization

## Competing Interests

The authors declare that they have no competing interests.

## Funding

This series was supported by sponsorship from the WHO.
