# Supplementary material for: Publishing data to support the fight against human vector-borne diseases
Source: Gigascience. 2022 Nov 3;11:giac114. doi: 10.1093/gigascience/giac114 (PMC9633277; doi:10.1093/gigascience/giac114)
Supplement: giac114_Supplemental_File [file giac114_supplemental_file.docx]

**Supplementary File 1: Interview questions with authors**

**Submission #1.**

Online catalogue of the Coleção de Flebotomíneos (FIOCRUZ/COLFLEB), a biological collection of American sand flies (Diptera: Psychodidae, Phlebotominae) held at Fiocruz Minas, Brazil

Paloma Helena Fernandes Shimabukuro, Alanna Silva Reis, Carolina Cunha Monteiro, Jose Dilermando Andrade Filho

Paper: <https://doi.org/10.46471/gigabyte.52>
Data: <https://doi.org/10.15468/sxcpfp>

**Can you summarise in a few sentences what your data represented, and highlight any interesting points about it?**

Our data is derived from approximately 72,000 individual specimens deposited in the Coleção de Flebotomíneos (FIOCRUZ/COLFLEB). Our data covers over 80 years of sand fly research in Brazil and other American countries which makes it the largest and most comprehensive collection of these insects. The data is published online through the speciesLink network and the Sistema de Informação sobre a Biodiversidade Brasileira (SiBBr) and it includes all of the available provenance information associated with each specimen. Our data can be used for several applications in epidemiology and control of leishamanises.

**Was there anything useful you and your co-authors got out of the process of putting these Data Release papers together?**

Yes, we´ve learned how to prepare a datapaper, how to maximize our collection´s data availability to a wider public.

**Submission #2.**

DRR-202202-04

Arbovirus vectors in municipalities with a high-risk of dengue in Cauca, southwestern Colombia.

Catalina Marcelo, Carlos Andres Morales, Maria Camila Lesmes, Patricia Fuya, Sergio Andres Mendez, Horacio Cadena, Alvaro Javier Avila, Erika Santamaria
Paper: <https://doi.org/10.46471/gigabyte.53>
Data: <https://doi.org/10.15472/dxbowv>

**Can you summarise in a few sentences what your data represented, and highlight any interesting points about it?**

We provided novel data for the geographical distribution of 2,383 specimens belonging to the Culicidae family present at Cauca, Colombia. Also, we report the house infestation percentage per municipality, house index and additional descriptive measures of the sampled mosquitos at each location. Differences between the Shannon and Simpson diversity indices per municipality were reported and all the mosquito data are available in the GBIF repository.

We would like to highlight the number of houses screened because adult Culicidae mosquitoes are not often reported, due to the sampling effort involved in this entomological sampling. Additionally, the sample of *Aedes albopictus* could involve an important epidemiological finding given its role as a secondary mosquito vector for dengue.

**Was there anything useful you and your co-authors got out of the process of putting these Data Release papers together?**

As entomologists and scientists, we learned about the importance of sharing collection data in repositories such as the GBIF, as well as disseminating the results of our studies in a concrete and prompt manner through "data release" publications, as this information is critical for decision making by stakeholders.

**Submission #3**

Mosquito Alert: Leveraging Citizen Science to Create a GBIF Mosquito Occurrence Dataset

Zivko Juznic Zonta, Isis Sanpera-Calbet, Roger Eritja, John Palmer, Agusti Escobar, Joan Garriga, Aitana Oltra, Alex Richter-Boix, Francis Schaffner, Alessandra della Torre, Miguel Angel Miranda, Marion Koopmans, Luisa Barzon, Frederic Bartumeus Ferre

Paper: <https://doi.org/10.46471/gigabyte.54>
Data: [https://doi.org/10.15470/t5a1os](https://doi.org/10.15470/t5a1os%20CC0) and <https://www.ebi.ac.uk/biostudies/studies/S-BIAD249>

**Can you summarise in a few sentences what your data represented, and highlight any interesting points about it?**

The Mosquito Alert dataset published on GBIF includes occurrence records of adult mosquitoes collected by citizens through the Mosquito Alert smartphone app. Each record is linked to a photograph which is validated by entomological experts to assess its species. The temporal coverage of the dataset is from 2014 through 2021 and the spatial coverage is worldwide. Most of the records from 2014 to 2020 are from Spain, while starting from 2020 the coverage increased in Europe, mainly in the Netherlands, Italy, and Hungary. Mosquito Alert is now a mature near-real-time surveillance system of five targeted disease-vector mosquito species of concern in the EU. From a surveillance perspective the system has been able to detect many appearances of *Aedes albopictus* much beyond its immediate expansion front, like in the Spanish Autonomous Communities of Andalucía and Aragón, which happened as well within other EU countries. A major highlight was the first detection in 2018 of *Aedes japonicus* in Spain, an isolated population located at 1,300 km distance of its nearest known location in Europe. The system also collaborated to assess the distribution area of the species across Northern Spain which was actually much broader than estimated in 2018. As a biodiversity feature, the expert validators have labeled as many as 28 autochtonous Culicid species throughout Europe in only two years. All these discoveries resulted in five publications ranging from 2014 to 2021.

**Was there anything useful you and your co-authors got out of the process of putting these Data Release papers together?**

We had to make many decisions on how to put together an organic, ever growing and mutating data collection system with many actors involved and highly heterogeneous contributions to the dataset by experts. We had to find the right format of author citation and set up a credit system to evaluate contributions from multiple and diverse collaborators. This exercise triggered many debates within Mosquito Alert team, and between Mosquito Alert team and the expert validation community, things going much beyond the dataset itself. Hopefully this work represents a big step towards the development of a system to give credit to the worldwide community of digital entomologists contributing to the Mosquito Alert dataset, and a better unfolding of the FAIR principles we aim to follow in the future.

**Submission #4.**

Culicidae (Diptera: Culicomorpha) in the southern Brazilian "Ana Leuch Lozovei" collection with notes on distribution and diversity

Andrey Andrade, Mauricio Conceicao, Samira Chahad-Ehlers, Luiz Santos-Neto, Adson SantAna, Gabriela Ribeiro, Debora Klisiowicz, Cassio Silva-Inacio, Taciano Moura, Reanata Gama, Ana Lozovei
Paper: <https://doi.org/10.46471/gigabyte.55>
Data: <https://doi.org/10.15468/g7628g>

**Can you summarise in a few sentences what your data represented, and highlight any interesting points about it?**

Our data represent an important contribution on the diversity of Culicidae in 18 municipalities in Paraná state, southern Brazil, collected between 1967 and 1999. The collection was an initiative of Professor Ana Leuch Lozovei and her students and collaborators who collected and identified most of the species that we organize, list and catalog here. In total, we have gathered 5,739 specimens with an incredible diversity of 100 species, of which 18 are new samples recorded for Paraná. Interestingly, we detected three species that are newly recorded samples for Brazil, signifying the expansion of geographical distribution of the species previously restricted to certain locations or countries.

**Was there anything useful you and your co-authors got out of the process of putting these Data Release papers together?**

One of the most useful things we got in the data-gathering process was when we started to associate species with the transmission of vector-borne diseases. This generated a list, shown in the original text, which emphasizes a significant number of specimens related to transmission of pathogens responsible for devastating diseases in humans and other animals, such as malaria, encephalitis and several viruses of great importance in current epidemics such as Yellow fever, Chikungunya, Zika and Dengue. Brazil has been suffering from deforestation and unsustainable agricultural practices that contribute greatly to climate change, which may alter vector habitats and consequently provide changes in the transmission patterns of infectious diseases. With a defined spatio-temporal distribution, ranging from forests on mountain slopes to peri-urban and urban regions, this Culicidae collection represents a potential reference for possible changes in the prevalence and geographic distribution of vector species for future entomological surveys.

**Submission #5.**

Tick abundance, diversity and pathogen data collected by the National Ecological Observatory Network

Sara H Paull, Kate Thibault, Abigail Benson
Paper: <https://doi.org/10.46471/gigabyte.56>
Data: <https://doi.org/10.15468/b52b9z>

**Can you summarise in a few sentences what your data represented, and highlight any interesting points about it?**

Our data represent 7 years of tick abundance, diversity and infection data collected using a standardized protocol by the National Ecological Observatory Network (NEON) at sites across the United States. The dataset is unique because of the availability of detailed surveillance (density) data alongside tick pathogen infection data across a large geographic area. An unprecedented level of associated environmental data collected at the same sites is also available from the NEON data portal, including: remote sensing data (e.g., NDVI and lidar), data on abiotic variables (e.g., temperature, humidity, precipitation), and observational data on nearby flora and fauna (e.g., small mammal populations, breeding birds and vegetation structure). These data are optimal for complex and multi-scale analyses of changing tick distributions, tick invasions and the patterns and processes underlying tick vector, host and pathogen dynamics. Archived samples of ticks included in the dataset add to its value and re-use potential.

**Was there anything useful you and your co-authors got out of the process of putting these Data Release papers together?**

Mapping our dataset to Darwin Core terms provided new insight into the contents of our dataset and linkages between ticks and pathogens. Providing a succinct overview of the utility of the dataset and the data collection and quality control methods helps us better communicate the strength of the data and potential questions for analysis.


**Submission #6**
AIMSurv: First pan-European harmonized surveillance of *Aedes* invasive mosquito species of relevance for human vector-borne diseases.
Paper: <https://doi.org/10.46471/gigabyte.57>
Data: <https://doi.org/10.15468/g7628g>

**Can you summarise in a few sentences what your data represented, and highlight any interesting points about it?**

AIMSurv presented data from the first pan-European harmonized surveillance of *Aedes* invasive mosquito species organized under the framework of the AIMCOST Action. A harmonized protocol was produced in 2020 setting up minimum requirements for a harmonized surveillance using ovitraps and adult traps of *Aedes* invasive mosquito (AIM) species in Europe. The protocol included a minimum sampling of three month, capturing the peak of abundance of AIM species in each country. The data gathered by AIMSurv represents the first collaborative (24 teams; 42 countries) surveillance of AIM species in Europe and will be the basic line to better understand the seasonal abundance of AIM species across Europe.

**Was there anything useful you and your co-authors got out of the process of putting these Data Release papers together?**

I think we should stress the high level of collaboration among all participants, since the AIMSurv activity was on the basis of free collaboration and data sharing. The series from *GigaByte* was an excellent opportunity spotted by Alessandra to publish all this collaborative activity in open-access and to be able to be shared with other vector surveillance initiatives in Europe and around the world. The data paper allowed to give proper recognition to the teams contributing to the AIMSurv data collection, in the form of author or collaborators of the AIMCOST-AIMSurv consortium. This is of very much interest for AIMCOST since data sharing and networking are priorities of the action and the *GigaByte* data paper provided the proper framework to achieve that.

**Submission #7.**

MODIRISK: Mosquito vectors of disease, collection, monitoring and longitudinal data from Belgium

Wim Van Bortel, Veerle Versteirt, Wouter Dekoninck, Thierry Hance, Dimitri Brosens, Guy Hendrickx

Paper: <https://doi.org/10.46471/gigabyte.58>
Data: Longitudinal dataset <https://doi.org/10.15468/rwsozv>; Inventory dataset <https://doi.org/10.15468/4fidg2>; Collection dataset <https://doi.org/10.15468/3in3fb>

and

**Submission #8:**

MEMO: Monitoring of Exotic MOsquitoes in Belgium

Dimitri Brosens, Isra Deblauwe, Katrien De Wolf, Nathalie Smitz, Adwine Vanslembrouck, Anna Schneider, Jacobus De Witte, Ingrid Verle, Wouter Dekoninck, Marc De Meyer, Thierry Backeljau, Sophie Gombeer, Kenny Meganck, Ann Vanderheyden, Ruth Muller, Wim Van Bortel
Paper: <https://doi.org/10.46471/gigabyte.59>

Data:<https://doi.org/10.15468/r42fr7> and<https://doi.org/10.15468/4u5aub>

**Can you summarise in a few sentences what your data represented, and highlight any interesting points about it?**

Dimi: For me, it is very interesting to dive into other colleagues' data and try to make something understandable for everyone out of it. It gives me insight on how colleagues try to deal with data and how we can transform this data in a standardized dataset using Darwin Core. It also creates the opportunity for me to learn about other biodiversity datasets.

Wouter: Data represented: detailed data of trap-samples of mosquitoes. Interesting here is that we were able to publish sampling data in such a detailed level. Other papers do not always allow that. Moreover the data become easily retrievable on-line and hence can be used for other studies too.

Guy: We actually came up with the MODIRISK idea and sampling strategy for spatial modelling and offered coordination to ITM because a private company was not allowed to coordinate a research project under BELSPO rules. This turned out to be the start of a great collaboration also expanding to other projects.

At the time MODIRISK was written little was known about mosquito species diversity and distribution in Belgium.

Especially the plan to model distributions at a 1km scale instead of simply recording species presence in grids was novel. We obtained funding for this project because in the early 2000’s decision makers became more concerned with the fact that there may be potential vectors waiting for the right pathogen in this part of the world too.

Wim: In fact it was the beginning of more work on mosquitoes, primarily exotic *Aedes* mosquitoes.

Isra: The data also gives the state-of-of-the-art of the exotic *Aedes* monitoring in Belgium. It is important as we are now at the beginning of the invasion process.

Nathalie: My data consists of DNA sequences submitted to GenBank and produced during MEMO:<https://www.ncbi.nlm.nih.gov/bioproject/837425>. This database will continue to be supplemented since we are still working on a few publications. DNA-barcoding was used as a quality control step by validating 5% of the morphological identifications of the yearly collections. This guarantees the quality of observations presented in the database, by validating the species identifications. Additionally, it helped to fine-tune some morphological species identifications for species/life stages lacking discriminative characters (ex. Culex pipiens and *Culex torrentium* – widespread in Belgium, or eggs) or species complexes (ex. Anopheles maculipennis s.l. – four native species are occurring in Belgium). So it brings some precisions in the observations reported in the database.

Veerle: Regarding the second question, I think Wim answered that it was the basis for all further research and as Guy wrote, the use of a randomised approach with the later Vecmap was a novel approach... So actually the project was not only very necessary but also very innovative.

The most important thing, for me anyway, was that with this approach we were not only able to detect most species but even a new exotic species (*Ochlerotatus/Aedes koreicus*) which had not been found in Europe until then (but then suddenly was when targeted).

**Was there anything useful you and your co-authors got out of the process of putting these Data Release papers together?**

Dimi: For me this is not the first datapaper I coordinated. What I mostly do is start a draft and let the specialists complete the draft. The final structure of the data release is somewhat different than the ones I coordinated before. (The others are more in line with the original GBIF metadata structure.)

Also, completing the data release paper and the datasets is always a learning process. In this case I learn about Mosquito & vector biodiversity, but I'm also able to 'teach/advocate' the co-authors about Open data, open science, data management, standards and data publication.

I'm happy that for the MeMo data release we could really include some links to genomic data. I thought that was really cool.

Wouter: It was good to trace back all the data and order them in a way which allows publishing them on-line.

Guy: I’m not sure what is meant here.

From our point of view data papers may turn out to become even more important than analysis papers. Data are driving science and should always be made freely available for any party to work with. Writing this paper contributed to that essential idea.

Wim: It took more time than initially foreseen, we started writing this paper in 2017. But the timing is good and I am happy that this data paper is out.

Isra: I think it is good to have these papers to refer too and also for other people to use the data.

Nathalie: I’m joining Guy’s idea: data papers are important to publicly present databases (to which one can also refer to) and stimulate further research.

Veerle: Preparing data papers allows you to critically review your plan of approach and the methods used in the light of the results obtained. It may allow you to gain new ideas and insights, which can lead to adjustments, improvements in the methodology and who knows, new projects/collaborations.

**Submission #9.**

Sand fly (Diptera: Psychodidae: Phlebotominae) records in Acre, Brazil: a Dataset

Rodrigo Espindola Godoy, Andrey Jose de Andrade, Paloma Helena Fernandes Shimabukuro, Andreia Fernandes Brilhante

Paper: <https://doi.org/10.46471/gigabyte.60>
Data: <https://doi.org/10.15468/c9arun>

**Can you summarise in a few sentences what your data represented, and highlight any interesting points about it?**

Our work makes a thorough review of all published scientific studies on sand flies in the state of Acre, located in the North region, Brazil. This is the state with the higher incidence of American cutaneous leishmaniasis in the Amazon region. In addition to presenting some new records. This is the first published dataset with information on these insects for the state that provide an important source of knowledge on the distribution, identification, and taxonomic status of the sand fly species already recorded. Which makes it a relevant material for future research in the area and for Amazon biome.

**Was there anything useful you and your co-authors got out of the process of putting these Data Release papers together?**

As the work was based on the review of literature, it was very enlightening to understand the chronology of how the researchers were carried out in the region and how the evolution in the knowledge of the diversity of sand flies in the state of Acre took place. Which later leads us to realise that many areas (municipalities) of the state remain without knowledge of the fauna of these insects. Showing that there is still a lot to be known in the state, in terms of the sand fly fauna. It was very helpful to learn how to make our data publicly available.

**Submission #10.**

Occurrence records and metadata for sand flies (Diptera, Psychodidae, Phlebotominae) collected in the lands of indigenous people in the Brazilian Amazon

Paloma Helena Fernandes Shimabukuro, Daniel Rocha Cangussu Alves, Jessica Adalia Costa Barros, Luiz Otavio Cordeiro Nascimento, Luke Baton, Maira Posteraro Freire, Manoel Edson Medeiros, Mauro Diego Gobira Guimaraes de Assis, Sofia Ferreira Morais, Tiago Silva da Costa, Veracilda Ribeiro Alves, Eduardo Stramandinoli Moreno
Paper: <https://doi.org/10.46471/gigabyte.61>
Data: <https://doi.org/10.15468/28xvr7> and <https://doi.org/10.15468/gt29ub>

**Can you summarise in a few sentences what your data represented, and highlight any interesting points about it?**

Our data comprises records of sand flies, insects involved in the transmission of leishmaniases, these records were obtained from areas of disease transmission in indigenous people lands in the Brazilian Amazon, where cutaneous leishmaniasis is endemic. We hope that our records will contribute to a better understanding of leishmaniasis transmission dynamics among indigenous people, as well as increase data on the distribution of insect vectors, in these areas.

**Was there anything useful you and your co-authors got out of the process of putting these Data Release papers together?**

Yes, we´ve learned how to prepare our datasets for publication through GBIF, and the importance of making such data open access.

**Submission #11**

Soledad Ceccarelli, Agustin Balsalobre, Maria Eugenia Vicente, Rachel Curtis-Robles, Sarah A Hamer, Jose Manuel Ayala Landa, Jorge E Rabinovich, Gerardo A Mart

American triatomine species occurrences: updates and novelties in the DataTri database
Paper: <https://doi.org/10.46471/gigabyte.62>
Data: <https://doi.org/10.15468/fbywtn>

**Can you summarise in a few sentences what your data represented, and highlight any interesting points about it?**

Our data represent a subdataset of American triatomine - insect vectors involved in Chagas disease – occurrences, as an update to the most complete and integrated database (DataTri) available to date at a continental scale. This work is the result of an exhaustive review of public information combined with substantial inter-institutional collaboration, which integrated information spanning 24 American countries. This geodatabase may contribute not only towards improving knowledge of the geographical distributions of every American triatomine species, but also to designing improved strategies for health promotion and vector control. We believe it will be of practical use for both the academic and educational community, as well as for those institutions responsible for public health promotion, prevention, and vector control activities. As the data is hosted in an open and public repository, we hope that they will contribute towards fulfilling national and international goals, such as promoting the exchange of biological information, increasing and improving the accessibility of such information, providing biological data produced and compiled in several countries, and enhancing knowledge of both the biodiversity and epidemiological data related to Chagas disease.

**Was there anything useful you and your co-authors got out of the process of putting these Data Release papers together?**

The process of putting the Data Release paper together was useful to establish an interesting collaborative work dynamic**.** In addition, it helped us to exchange knowledge about data management, understand more about how we might expand our contributions to the database efforts and learn more about our respective community science programs from USA (Kissing Bugs and Chagas Disease in the United States) and Argentina (GeoVin).
